# Association between frailty and chest pain: Insights from the 2009 to 2018 NHANES cross-sectional analysis and Mendelian randomization

**DOI:** 10.1097/MD.0000000000044517

**Published:** 2025-09-19

**Authors:** Zhe Zhang, Mengting Chen, Dong Cai, Shushen Weng, Lingling Zhao, Yang Wang

**Affiliations:** a Department of Traditional Internal Medicine, The Affiliated People’s Hospital of Fujian University of Traditional Chinese Medicine, Fuzhou, Fujian, China; b School of Traditional Chinese Medicine, Fujian University of Traditional Chinese Medicine, Fuzhou, Fujian, China.

**Keywords:** cardiovascular risk, chest pain, frailty, Mendelian randomization, NHANES

## Abstract

Chest pain is a common clinical symptom with diverse etiologies, including cardiovascular, respiratory, digestive, and musculoskeletal disorders, and is one of the leading causes of emergency department visits. However, the association between frailty and chest pain has not been well characterized. We analyzed 1019 participants from the 2009 to 2018 National Health and Nutrition Examination Survey. Frailty was assessed using a 49-item frailty index (FI), evaluated as both a continuous variable and a categorical variable (frail: FI ≥ 0.21; non-frail: FI < 0.21). Chest pain was identified from a self-reported item in the National Health and Nutrition Examination Surve Health questionnaire, without distinguishing cardiac from noncardiac causes. Weighted logistic regression models were applied: model 1 (unadjusted), model 2 (adjusted for demographic variables), and model 3 (further adjusted for socioeconomic, lifestyle, medication use, clinical conditions, and metabolic markers). Sensitivity analyses included subgroup and interaction analyses, unweighted models, and multiple imputation. A 2-sample Mendelian randomization analysis, using genome-wide association study summary statistics, was conducted to explore potential causality. After adjusting for all covariates, FI was significantly associated with chest pain (odds ratio [OR] = 4.69, 95% confidence interval [CI]: 2.18–10.08, *P* < .001). In subgroup analyses, this association was significant in the frail population (OR = 2.78, 95% CI: 1.27–6.09, *P* = .017), but not in the non-frail group. Sensitivity analyses confirmed the robustness of these findings. Mendelian randomization analysis further indicated a potential causal relationship between FI and chest pain (inverse variance weighting OR = 1.12, 95% CI: 1.10–1.14, *P* < .001). Frailty is independently associated with self-reported chest pain, and genetic analyses suggest a possible causal relationship. However, these findings should be interpreted with caution, as chest pain may originate from multiple underlying conditions.

## 1. Introduction

Chest pain is a prevalent clinical symptom with diverse etiologies, including cardiovascular, respiratory, digestive, and musculoskeletal disorders, and affects an estimated 20% to 40% of individuals throughout their lifetime.^[1,2]^ It is also among the most frequent reasons for emergency department visits.^[3–5]^ In 2017, data from the National Center for Health Statistics (NCHS) indicated that chest pain was responsible for 4.7% of all emergency department visits, amounting to 6.523 million cases, making it the second most common complaint after abdominal pain (8.8%).^[6]^ Chest pain is generally categorized into cardiac and noncardiac causes,^[5]^ with over half of the cases being of noncardiac origin.^[7]^ Thus, early identification of risk factors for chest pain is crucial for timely intervention and improved clinical outcomes.

Frailty is a clinical syndrome defined by the gradual deterioration of physiological systems caused by stressors and impaired homeostasis.^[8]^ It is commonly characterized by diminished gait speed, fatigue, reduced grip strength, lower physical activity, and unintended weight loss.^[9]^ The prevalence of frailty is projected to increase as the population continues to age.^[10]^ Research suggests that in individuals aged ≥50 years, frailty prevalence ranges from 12% to 24%, whereas pre-frailty affects an estimated 46% to 49%.^[11]^ Numerous studies have demonstrated that frailty is associated with a wide spectrum of adverse health outcomes, including cardiovascular diseases and their complications, respiratory diseases, metabolic disorders, and pain.^[12–16]^ However, direct clinical evidence linking frailty specifically to chest pain remains scarce. This knowledge gap underlines the importance of further investigation.

The National Health and Nutrition Examination Survey (NHANES) (https://www.cdc.gov/nchs/nhanes/) is a nationally representative cross-sectional survey conducted by the Centers for Disease Control and Prevention (CDC) to evaluate the nutritional and health status of adults and children in the United States.^[17]^ It provides a comprehensive dataset that enables the evaluation of the relationship between frailty and chest pain. However, observational studies frequently suffer from residual confounding.^[18]^ Mendelian randomization (MR) is a powerful analytical approach for determining causal relationships between risk factors and disease outcomes.^[19]^ This approach utilizes genetic variants as instrumental variables (IVs) to estimate causal effects, thereby minimizing bias due to confounding factors.^[20]^

Therefore, this study seeks to examine the association between frailty and self-reported chest pain using NHANES cross-sectional data and to explore potential causality through MR analysis.

## 2. Materials and Methods

### 2.1. Cross-sectional study design and data sources and processing

**Figure 1.**
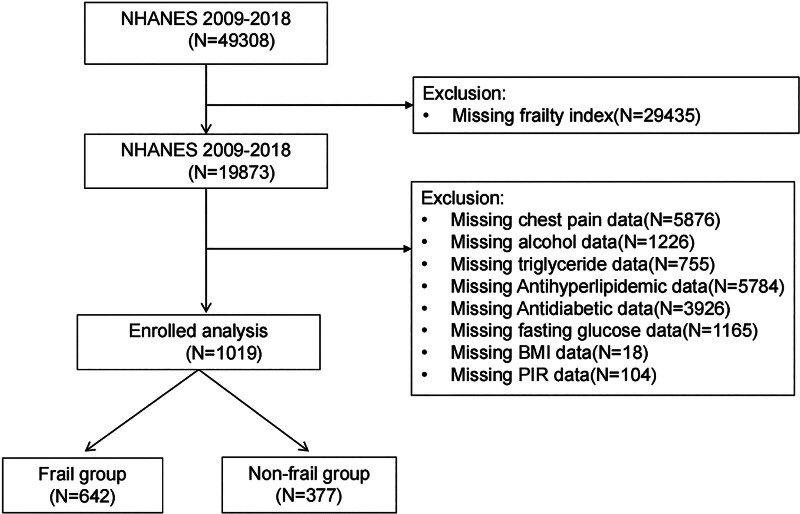
Flowchart of participant selection. BMI = body mass index, NHANES = National Health and Nutrition Examination Survey, PIR = poverty income ratio.

The NHANES study was approved by the Institutional Review Board of the NCHS, and informed consent was obtained from all participants.^[21]^ This study analyzed data from 5 NHANES survey cycles (2009–2018), encompassing a total of 49,308 participants. To ensure data integrity and completeness, participants with missing values were excluded based on predefined criteria, as detailed in Figure 1. Specifically, 29,435 participants missing frailty index (FI) data, 5876 missing chest pain data, 1226 missing alcohol intake information, 755 missing triglyceride (TG) values, 5784 missing antihyperlipidemic medication use data, 3926 missing antidiabetic medication use data, 1165 missing fasting glucose (FG) measurements, 18 missing body mass index (BMI) values, and 104 missing poverty income ratio (PIR) information were excluded. After applying these exclusion criteria, a total of 1019 participants with complete datasets were included in the final analysis. Participants were then categorized into the frail group (FI ≥ 0.21) and the non-frail group (FI < 0.21) based on their FI, facilitating a more detailed examination of the relationship between frailty and chest pain.

### 2.2. Variables included in the cross-sectional analysis

#### 2.2.1. Selection of frailty and chest pain

Frailty was designated as the primary exposure variable in this study. The FI was assessed using 49 indicators to evaluate the cumulative burden of physiological deficits (Table S1, Supplemental Digital Content, https://links.lww.com/MD/P990). To ensure data completeness and reliability, only participants who responded to at least 80% (≥39 items) of the FI indicators were included in the analysis.^[22]^ The FI was computed as the ratio of reported deficits to the total number of items answered. Based on the predefined threshold of 0.21, participants were classified as frail (FI ≥ 0.21) or non-frail (FI < 0.21).^[23–25]^

Chest pain was defined as the primary outcome variable. Participants were categorized as experiencing chest pain if they answered “yes” to the NHANES Health questionnaire item CDQ001^[26]^: “{Have you/Has SP} ever experienced any pain or discomfort in {your/her/his} chest?.” This item captures any chest pain or discomfort of diverse possible origins – including cardiovascular, respiratory, gastrointestinal, and musculoskeletal causes – and is therefore a self-reported symptom measure rather than a confirmed clinical diagnosis.

#### 2.2.2. Selection of other covariates

The covariates included in this study covered multiple domains: demographic factors (gender, age, race, education level, PIR); lifestyle behaviors (alcohol consumption status, smoking status, vigorous physical activity); clinical conditions (hypertension, heart failure, coronary heart disease [CHD], angina pectoris, heart attack, stroke, type 2 diabetes mellitus [T2DM]); metabolic markers (total cholesterol [TC], TG, FG, BMI); as these medications may influence both frailty status and chest pain risk and thus were adjusted for as potential confounders.^[26–28]^

Following WHO guidelines, BMI was classified into 3 categories: <25 kg/m² (normal weight), 25 to 29.9 kg/m² (overweight), and ≥30 kg/m² (obese).^[29]^ The PIR was stratified into 3 economic strata: low-income (PIR ≤ 1), middle-income (PIR between 1 and <4), and high-income (PIR ≥ 4).^[30]^

The diagnoses of T2DM was defined as^[17,29]^: diagnosis by a doctor or other health professional; HbA1c level ≥ 6.5 mmol/L; fasting plasma glucose ≥126 mg/dL; taking insulin now; current use of antidiabetic medications.

Hypertension was diagnosed according to recognized clinical guidelines,^[31]^ which included: a diagnosis by a physician or healthcare professional; systolic blood pressure ≥130 mm Hg and/or diastolic blood pressure ≥80 mm Hg; current use of antihypertensive medications.

### 2.3. Data sources and choice of instrumental variables for Mendelian randomization

Table S2, Supplemental Digital Content, https://links.lww.com/MD/P990 outlines the exposure and outcome genome-wide association study (GWAS) datasets used in the MR analysis. All datasets are publicly available, thus no additional ethical approval was required.

- Exposure (frailty): single nucleotide polymorphisms (SNPs) associated with frailty were obtained from a large GWAS meta-analysis by Atkins et al^[32]^ (phenotype code: GCST90020053), comprising 1,75,226 individuals of European ancestry (1,64,610 from the United Kingdom and 10,616 from Sweden) and 76,63,023 SNPs.
- Outcome (chest pain): GWAS summary statistics for chest pain were obtained from the UK Biobank (https://www.nealelab.is/uk-biobank; phenotype code: 2335), including 5,01,260 European participants and 13,770,979 SNPs.

**Figure 2.**
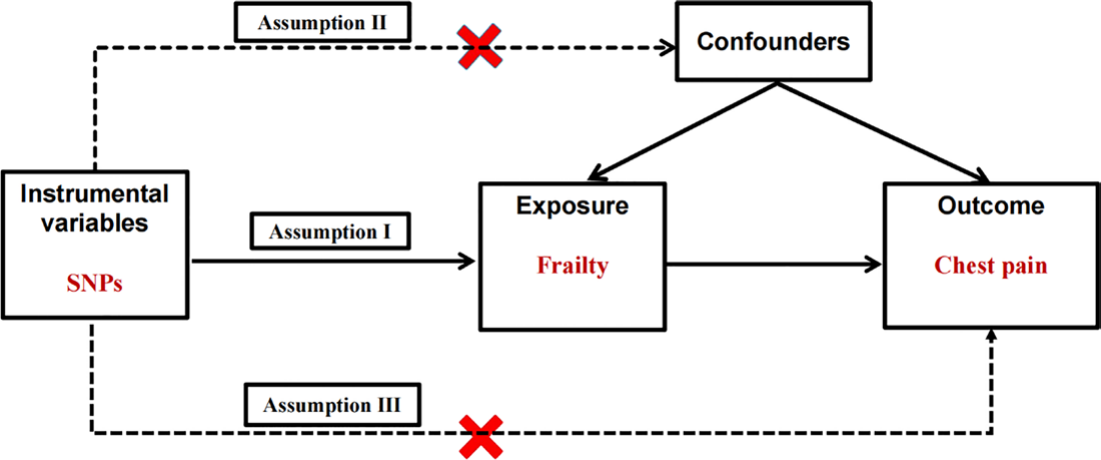
Overall design of Mendelian randomization study. Red crosses indicate that the pathway is not allowed. SNPs = single nucleotide polymorphisms.

IV selection criteria: In line with the 3 core MR assumptions (Fig. 2)^[33]^ – the IV is robustly associated with the exposure; the IV is independent of confounders; and the IV influences the outcome only through the exposure – SNPs were selected as follows:

- Genome-wide significance threshold of *P* < 5 × 10⁻⁷.
- Linkage disequilibrium pruning with *r*² < 0.001 within a 10,000 kb window.
- Instrument strength evaluated using an *F*-statistic > 10, calculated as *F* = β²/ SE², to ensure robustness.^[34]^

### 2.4. Statistical methods

All cross-sectional analyses were performed using R (version 4.4.1). NHANES data from five 2-year cycles (2009–2018) were analyzed with appropriate survey weights. Original 2-year weights were divided by 5 to account for the 10-year analytic period.^[35,36]^ Continuous variables (mean ± SD) were compared using *T*-tests, and categorical variables (N, %) using chi-square tests. Variance inflation factors (VIFs) < 5 indicated no severe multicollinearity.^[37,38]^ Weighted logistic regression models estimated associations between frailty and chest pain, and results are presented as odds ratios (ORs) with 95% confidence intervals (CIs):

- Model 1: unadjusted.
- Model 2: adjusted for demographic variables (age, sex, race, education).
- Model 3: further adjusted for socioeconomic (PIR), lifestyle (alcohol, smoking, physical activity), medication use (antihyperlipidemic, antidiabetic), clinical conditions and metabolic measures (hypertension, heart failure, CHD, angina, myocardial infarction, stroke, T2DM, TC, TG, FG, BMI).

Restricted cubic spline (RCS) models were used to assess nonlinearity. Sensitivity analyses included: subgroup and interaction analyses; analyses repeated using unweighted data; and analyses repeated after multiple imputation.

MR analyses were conducted using R software (version 4.4.1) with the packages “Two-Sample-MR,” “MR-PRESSO,” and “Mendelian Randomization.” Initially, MR-PRESSO was used to detect and exclude potential outliers.^[39]^ Cochrane’s *Q* statistic was employed to assess heterogeneity among SNPs.^[40]^ A random-effects model was applied if significant heterogeneity was detected (*P* < .05); otherwise, a fixed-effects model was utilized.^[41]^ Heterogeneity was further visualized using funnel plots.^[21]^ Subsequently, 3 MR analytical methods – inverse variance weighting (IVW),^[42]^ MR-Egger regression,^[43]^ and weighted median (WM) estimation^[44]^ – were applied to infer the causal relationship between frailty and chest pain, with IVW serving as the primary analytical method.^[45,46]^ Additionally, MR-Egger intercept tests were performed to detect horizontal pleiotropy.^[44]^ To assess the robustness of the results, leave-one-out sensitivity analyses were performed, whereby influential SNPs were identified, excluded, and MR estimates were recalculated with the remaining SNPs.^[47]^ A *P* value of <.05 was considered statistically significant.

## 3. Results

### 3.1. Baseline characteristics of NHANES participants

Table S3, Supplemental Digital Content, https://links.lww.com/MD/P990 summarizes the baseline characteristics of the 1019 participants included in the study. Participants were categorized based on the presence (n = 366) or absence (n = 653) of chest pain. Among these participants, 531 (52.1%) were female, and 488 (47.9%) were male. According to the FI, 642 (63.0%) participants were classified as frail (FI ≥ 0.21), and 377 (37.0%) as non-frail (FI < 0.21). Participants reporting chest pain exhibited significantly higher FI compared to those without chest pain (0.31 vs 0.23, *P* < .001), a pattern similarly evident within the frail subgroup (0.35 vs 0.31, *P* < .001). Furthermore, cardiovascular diseases were significantly more prevalent among participants experiencing chest pain, including CHD (overall: 22.7% vs 6.4%; frail subgroup: 25.3% vs 9.5%), angina pectoris (overall: 19.1% vs 2.5%; frail subgroup: 22.2% vs 4.6%), and heart attack (overall: 23.2% vs 4.9%; frail subgroup: 27.0% vs 7.4%). Additionally, a higher proportion of low-income individuals was observed in the chest pain group compared to the non-chest pain group (overall: 29.0% vs 24.2%; frail subgroup: 31.7% vs 28.7%, *P* < .05). VIF analysis showed that all covariates had VIF values below 10, indicating no significant multicollinearity. Detailed VIF results are provided in Table S4, Supplemental Digital Content, https://links.lww.com/MD/P990.

### 3.2. Associations between frailty index, frail status, and non-frail status with chest pain

**Figure 3.**
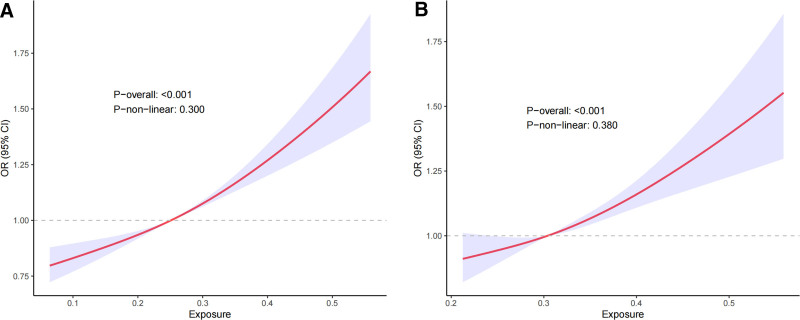
Restricted cubic spline analysis assessing the associations between frailty index (A), frail status (B), and chest pain. The associations were adjusted for age, sex, race, PIR, education level, BMI, smoking status, alcohol intake status, hypertension, TG, TC, FG, vigorous physical activity, heart failure, coronary heart disease, angina pectoris, heart attack, stroke, and T2DM, antihyperlipidemic and antidiabetic medications. BMI = body mass index, CI = confidence interval, CHD = coronary heart disease, FG = fasting glucose, OR = odds ratio, PIR = poverty income ratio, T2DM = type 2 diabetes mellitus, TC = total cholesterol, TG = triglyceride.

Table 1 presents the associations between quartiles of the FI, frail status, non-frail status, and the risk of chest pain. In model 1, without adjustments, the incidence of chest pain increased significantly with higher FI quartiles (OR = 6.01, 95% CI: 3.12–11.61, *P* < .001). After adjusting for key demographic variables (age, sex, race, PIR, and education level) in model 2, the FI remained significantly associated with chest pain (OR = 6.13, 95% CI: 3.11–12.06, *P* < .001). In model 3, after further adjustments for BMI, smoking status, alcohol intake status, hypertension, TC, TG, FG, vigorous physical activity, antihyperlipidemic and antidiabetic medication use, and clinical conditions including heart failure, CHD, angina pectoris, heart attack, stroke, and T2DM, the association between FI and chest pain remained significant, although the strength of the association slightly decreased (OR = 4.69, 95% CI: 2.18–10.08, *P* < .001). The stratified analyses by frailty status showed distinct patterns. Within the frail group, chest pain incidence also increased with higher FI quartiles. In model 1, the chest pain risk in Q4 was 3.55 times higher compared to Q1 (OR = 3.55, 95% CI: 1.81–6.98, *P* < .001). After demographic adjustments in model 2, the OR slightly increased to 3.69 (95% CI: 1.87–7.28, *P* < .001). However, in model 3, after additional adjustments for health conditions and lifestyle variables, the OR decreased to 2.78 (95% CI: 1.27–6.09, *P* = .017), but the association remained statistically significant. Conversely, in the non-frail group, no statistically significant association between FI and chest pain was observed in any of the models. In model 1, the OR comparing Q4 to Q1 was 1.84 (95% CI: 0.63–5.39, *P* = .274). After demographic adjustments in model 2, the OR slightly increased to 2.01 (95% CI: 0.66–6.06, *P* = .227) but remained nonsignificant. Even with further adjustments for health conditions and lifestyle factors in model 3, the OR for Q4 was 3.35 (95% CI: 0.83–13.48, *P* = .115), and statistical significance was still not reached. Given the significant positive associations identified between FI, frailty status, and chest pain, we further investigated potential nonlinear relationships using RCS analysis. The RCS analysis revealed linear relationships between FI, frailty status, and chest pain (Fig. 3A, B, *P* > .05).

**Table 1:** Associations between frailty index, frail status, and non-frail status and chest pain.

Exposure	Model 1 OR (95% CI) [*P* value]	Model 2 OR (95% CI) [*P* value]	Model 3 OR (95% CI) [*P* value]
Frailty index			
Quartiles			
Q1	Reference	Reference	Reference
Q2	1.94 (0.95–3.95) [*P* *=* .074]	1.98 (0.97–4.05) [*P* = .067]	1.86 (0.87–3.98) [*P* = .120]
Q3	2.61 (1.26–5.39) [*P* = .012]	2.67 (1.29–5.53) [*P* = .011]	2.25 (1.06–4.75) [*P* = .043]
Q4	6.01 (3.12–11.61) [*P* < .001]	6.13 (3.11–12.06) [*P* < .001]	4.69 (2.18–10.08) [*P* < .001]
Frail status			
Quartiles			
Q1	Reference	Reference	Reference
Q2	1.10 (0.63–1.91) [*P* *=* .734]	1.10 (0.61–1.98) [*P* = .745]	1.38 (0.74–2.60) [*P* *=* .323]
Q3	1.32 (0.78–2.22) [*P* = .307]	1.33 (0.80–2.22) [*P* = .273]	1.61 (0.95–2.76) [*P* *=* .091]
Q4	3.55 (1.81–6.98) [*P* < .001]	3.69 (1.87–7.28) [*P* < .001]	2.78 (1.27–6.09) [*P* *=* .017]
Non-frail status			
Quartiles			
Q1	Reference	Reference	Reference
Q2	1.88 (0.66–5.35) [*P* *=* .242]	1.61 (0.57–4.54) [*P* *=* .372]	2.56 (0.66–9.99) [*P* *=* .200]
Q3	1.48 (0.46–4.80) [*P* *=* .517]	1.41 (0.46–4.29) [*P* *=* .550]	2.21 (0.63–7.81) [*P* *=* .242]
Q4	1.84 (0.63–5.39) [*P* = .274]	2.01 (0.66–6.06) [*P* *=* .227]	3.35 (0.83–13.48) [*P* *=* .115]

Model 1: Unadjusted model.

Model 2: Adjusted for age, sex, race, PIR, and education level.

Model 3: Adjusted for age, sex, race, PIR, education level, BMI, smoking status, alcohol intake status, hypertension, TG, TC, FG, vigorous physical activity, heart failure, coronary heart disease, angina pectoris, heart attack, stroke, and T2DM, antihyperlipidemic and antidiabetic medications.

BMI = body mass index, CI = confidence interval, FG = fasting glucose, OR = odds ratio, PIR = poverty income ratio, T2DM = type 2 diabetes mellitus, TC = total cholesterol, TG = triglyceride.

### 3.3. Sensitivity analysis

**Figure 4.**
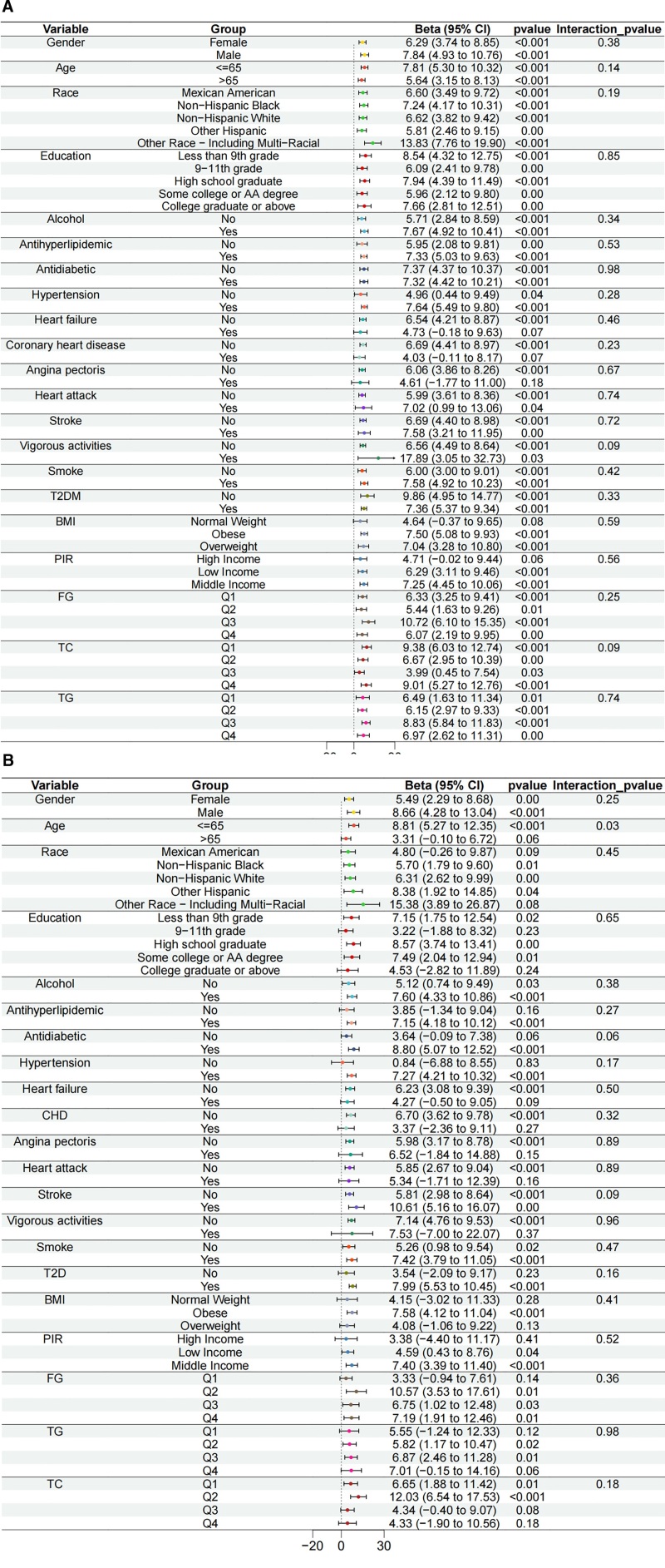
Subgroup analysis of the association between frailty index (A), frail status (B), and chest pain. Stratified by age, sex, race, PIR, education level, BMI, smoking status, alcohol intake status, hypertension, TG, TC, FG, vigorous physical activity, heart failure, coronary heart disease, angina pectoris, heart attack, stroke, T2DM, antihyperlipidemic and antidiabetic medications. BMI = body mass index, CI = confidence interval, CHD = coronary heart disease, FG = fasting glucose, OR = odds ratio, PIR = poverty income ratio, TC = total cholesterol, TG = triglyceride, T2DM = type 2 diabetes mellitus.

Subgroup and interaction analyses were subsequently performed to identify potential moderators of the frailty–chest pain association. No significant interactions between FI and covariates were found overall (Fig. 4A). However, within the frail subgroup, age demonstrated a statistically significant interaction (Fig. 4B), suggesting that age might modify the relationship between frailty and chest pain, while other clinical characteristics showed minimal impact.

In addition, no significant differences were observed in baseline characteristics before and after multiple imputation (Table S5, Supplemental Digital Content, https://links.lww.com/MD/P990), indicating that the imputation process had minimal impact on the data structure. Analyses based on both the unweighted dataset and the multiply imputed dataset consistently showed that the associations between the FI, frail status, and non-frail status with chest pain remained largely unchanged (Tables S6 and S7, Supplemental Digital Content, https://links.lww.com/MD/P990), thereby supporting the robustness of the study findings.

### 3.4. Causal relationship between frailty and chest pain

According to our predefined criteria, 31 eligible SNPs with *F*-statistics >10 were selected to evaluate the causal relationship between frailty and chest pain (Table S8, Supplemental Digital Content, https://links.lww.com/MD/P990). The forest plot showed a consistent positive association between genetically predicted frailty and chest pain for both individual SNPs and the combined analysis (Fig. S1A, Supplemental Digital Content, https://links.lww.com/MD/P993). MR-PRESSO analysis detected no significant outliers among these SNPs (*P* = .87; Table S9, Supplemental Digital Content, https://links.lww.com/MD/P990). Moreover, Cochran’s *Q* test indicated low heterogeneity among the SNPs (Table S9, Supplemental Digital Content, https://links.lww.com/MD/P990), and funnel plot visualization further supported minimal heterogeneity (Fig. S1B, Supplemental Digital Content, https://links.lww.com/MD/P993). Given this low heterogeneity, a fixed-effects model was used for subsequent MR estimation. The IVW results provided strong evidence for a causal relationship, demonstrating that genetically predicted frailty significantly increased the risk of chest pain (OR = 1.12, 95% CI: 1.10–1.14, *P* < .001; Table 2, Fig. S1C, Supplemental Digital Content, https://links.lww.com/MD/P993). Furthermore, the MR-Egger intercept test confirmed the absence of significant horizontal pleiotropy (*P* = .16; Table S9, Supplemental Digital Content, https://links.lww.com/MD/P990). Finally, leave-one-out sensitivity analyses revealed that no single SNP significantly influenced the overall MR results (Fig. S1D, Supplemental Digital Content, https://links.lww.com/MD/P993), indicating the robustness and reliability of the causal inference.

**Table 2:** Results of the causality analysis between frailty and chest pain.

Causal relationship	SNP	Method	OR (95%)	*P*
Frailty and chest pain	31	IVW	1.12 (1.10,1.14)	<.001
		MR Egger	1.06 (0.99,1.15)	.11
		Weighted median	1.11 (1.08,1.14)	<.001

IVW = inverse variance weighted, MR = Mendelian randomization, OR = odds ratio, SNP = single nucleotide polymorphism.

## 4. Discussion

This study leveraged nationally representative cross-sectional data from NHANES (2009–2018) together with 2-sample MR to examine, for the first time to our knowledge, the relationship between frailty and chest pain at the symptom level. Higher FI scores were consistently associated with greater odds of chest pain after extensive adjustment for demographic, lifestyle, clinical, and metabolic factors. When participants were stratified by frailty status, the association was evident in the frail subgroup but not in the non-frail subgroup, indicating that symptom susceptibility concentrates among individuals with clinically meaningful frailty. Complementary MR analyses further showed that genetically predicted frailty increases the risk of chest pain, supporting a causal contribution of frailty to the occurrence of this symptom.

Several previous studies have examined the association between frailty and chest pain or pain-related symptoms from different perspectives. Huang et al^[48]^ used longitudinal data from large national aging cohorts in China, the United Kingdom, and the United States and found that frailty significantly increased the risk of trunk pain – including chest, abdominal, back, and lumbar pain – with consistent associations across countries. However, chest pain was not analyzed as an independent outcome, and younger populations were not included. Yang et al^[49]^ conducted a systematic review and meta-analysis of 33 studies involving 25,900 older adults aged 60 years or above, demonstrating that chronic pain was associated with an increased risk of frailty. Nevertheless, most outcomes were defined as broad musculoskeletal pain, and chest pain was not explicitly examined as a distinct endpoint. Genetic studies have offered additional insight into the frailty–pain relationship. Zhong et al^[50]^ employed bidirectional MR and identified a causal relationship between genetically predicted frailty and multiple-site chronic pain, predominantly affecting the joints, limbs, and back, but again, chest pain was not directly evaluated. Li et al^[51]^ provided genetic evidence that frailty increases the risk of coronary artery disease and myocardial infarction, indirectly suggesting that frailty might elevate the risk of cardiac-related chest pain, although their analysis focused on disease endpoints rather than symptom level outcomes.

Compared with these studies, our work specifically focuses on chest pain as a symptom and offers several novel contributions. First, in our study, chest pain was defined using NHANES item CDQ001 from the Cardiovascular Health questionnaire, which asks whether participants have “ever had any pain or discomfort in the chest,” without specifying the cause. While this definition lacks etiological specificity, it intentionally captures a broad spectrum of real-world chest pain experiences – including both cardiac and noncardiac etiologies – rather than limiting the definition to adjudicated cardiovascular diagnoses. Such a symptom-centered perspective is clinically meaningful given the frequent presentation of chest pain in both primary care and specialist settings. Second, by analyzing chest pain as a standalone symptom and applying a consistent questionnaire definition, we provide more direct symptom level evidence than previous studies that grouped chest pain within “trunk pain” or broad chronic pain categories. Third, to our knowledge, this is the first study to apply 2-sample MR to the frailty–chest pain association, adding genetic causal inference to observational associations and filling a critical gap in prior literature where causal pathways were not addressed.

Several mechanisms could explain how frailty contributes to chest pain. For cardiovascular etiologies, potential pathways include chronic systemic inflammation,^[52,53]^ endothelial dysfunction,^[54]^ and autonomic dysregulation,^[55–57]^ all of which are more prevalent in frail individuals and can lower the threshold for chest discomfort. For non-cardiovascular etiologies, frailty may predispose individuals to respiratory compromise,^[14,15,58]^ gastrointestinal motility disorders,^[59–61]^ or musculoskeletal pain,^[48,62]^ any of which may contribute to chest pain.^[2]^ These pathways underscore the complex, multisystem nature of the frailty–chest pain relationship.

Several limitations should be acknowledged. First, although our symptom definition was chosen to maximize inclusivity and real-world relevance, chest pain was self-reported and did not differentiate between cardiac and noncardiac causes, which may introduce heterogeneity into the outcome definition. Second, although our mechanistic discussion considered both cardiovascular and non-cardiovascular pathways, we did not directly test these mechanisms. Third, our observational data were derived from the United States, whereas the MR data were based on European cohorts, which may limit generalizability across ethnic groups. Finally, despite adjustment for a wide range of confounders, residual and unmeasured confounding cannot be completely excluded.

## 5. Conclusion

In conclusion, frailty may be an independent risk factor for self-reported chest pain or discomfort. Given that the NHANES chest pain measure does not distinguish between cardiac and noncardiac causes, our findings should be interpreted cautiously at the symptom level. Early identification of frailty and appropriate interventions may help prevent the occurrence of chest pain from various etiologies.

## Acknowledgments

We thank all the authors who participated in this study. We thank the NHANES database (https://www.cdc.gov/nchs/nhanes/), UKB database (https://www.nealelab.is/uk-biobank) and GWAS Catalog database (https://www.ebi.ac.uk/gwas/home), for providing summary level data and the efforts of all the researchers.

## Author contributions

**Conceptualization:** Zhe Zhang.

**Data curation:** Mengting Chen, Dong Cai, Shushen Weng, Lingling Zhao.

**Formal analysis:** Zhe Zhang, Mengting Chen, Dong Cai, Shushen Weng, Lingling Zhao.

**Investigation:** Mengting Chen, Dong Cai, Shushen Weng, Lingling Zhao.

**Methodology:** Zhe Zhang.

**Supervision:** Yang Wang.

**Writing – original draft:** Zhe Zhang.

**Writing – review & editing:** Yang Wang.

## Supplementary Material


