# Supplementary material for: Association between frailty and chest pain: Insights from the 2009 to 2018 NHANES cross-sectional analysis and Mendelian randomization
Source: Medicine (Baltimore). 2025 Sep 19;104(38):e44517. doi: 10.1097/MD.0000000000044517 (PMC12459587; doi:10.1097/MD.0000000000044517)
Supplement: Supplementary file 1 [file medi-104-e44517-s001.pdf]

Supplementary Table S1.Variables in Frailty Index and Their Respective Scores

Items	scores
**Cognition**	
1.experience confusion/memory problems	yes=1, no=0
**Dependence**	
2.managing money difficulty	no difficulty=0, Some difficulty=0.33, much difficulty=0.66, unable to do=1
3.walking for a quarter mile difficulty	no difficulty=0, Some difficulty=0.33, much difficulty=0.66, unable to do=1
4.walking up ten steps difficulty	no difficulty=0, Some difficulty=0.33, much difficulty=0.66, unable to do=1
5.stooping, crouching, kneeling difficulty	no difficulty=0, Some difficulty=0.33, much difficulty=0.66, unable to do=1
6.lifting or carrying difficulty	no difficulty=0, Some difficulty=0.33, much difficulty=0.66, unable to do=1
7.house chore difficulty	no difficulty=0, Some difficulty=0.33, much difficulty=0.66, unable to do=1
8.preparing meals difficulty	no difficulty=0, Some difficulty=0.33, much difficulty=0.66, unable to do=1
9.standing up from armless chair difficulty	no difficulty=0, Some difficulty=0.33, much difficulty=0.66, unable to do=1
10.getting in and out of bed difficulty	no difficulty=0, Some difficulty=0.33, much difficulty=0.66, unable to do=1
11.using fork, knife, drinking from cup difficulty	no difficulty=0, Some difficulty=0.33, much difficulty=0.66, unable to do=1
12.dressing yourself difficulty	no difficulty=0, Some difficulty=0.33, much difficulty=0.66, unable to do=1
13.standing for long periods difficulty	no difficulty=0, Some difficulty=0.33, much difficulty=0.66, unable to do=1
14.grasp/holding small objects difficulty	no difficulty=0, Some difficulty=0.33, much difficulty=0.66, unable to do=1
15.attending social event difficulty	no difficulty=0, Some difficulty=0.33, much difficulty=0.66, unable to do=1
16.leisure activity at home difficulty	no difficulty=0, Some difficulty=0.33, much difficulty=0.66, unable to do=1
17.push or pull large objects difficulty	no difficulty=0, Some difficulty=0.33, much difficulty=0.66, unable to do=1
**Depressive Conditions**	
18.have little interest in doing things	nearly every day = 1, more than half the days = 0.66, several days = 0.33, no =0
19.feeling down, depressed, or hopeless	nearly every day = 1, more than half the days = 0.66, several days = 0.33, no =0

20.trouble sleeping or sleeping too much	nearly every day = 1, more than half the days = 0.66, several days = 0.33, no =0
21.feeling tired or having little energy	nearly every day = 1, more than half the days = 0.66, several days = 0.33, no =0
22.poor appetite or overeating	nearly every day = 1, more than half the days = 0.66, several days = 0.33, no =0
23.feeling bad about yourself	nearly every day = 1, more than half the days = 0.66, several days = 0.33, no =0
24.trouble concentrating on things	nearly every day = 1, more than half the days = 0.66, several days = 0.33, no =0
**Comorbidities**	
25.doctor ever said you had arthritis	yes = 1, no = 0
26.ever told you had thyroid problem	yes = 1, no = 0
27.ever told you had chronic bronchitis	yes = 1, no = 0
28.ever told you had cancer or malignancy	yes = 1, no = 0
29.ever told had congestive heart failure	yes = 1, no = 0
30.ever told you had coronary heart disease	yes = 1, no = 0
31.ever told you had angina/angina pectoris	yes = 1, no = 0
32.ever told you had heart attack	yes = 1, no = 0
33.ever told you had a stroke	yes = 1, no = 0
34.ever told you had high blood pressure	yes = 1, no = 0
35.doctor told you have diabetes	yes = 1, borderline=0.5, no =0
36.ever told you had weak/failing kidneys	yes = 1, no =0
37.urine leakage bother you?	greatly = 1, very much =0.75, somewhat= 0.5, only a little = 0.25, no=0
**Hospital and Care**	
38.general health condition	excellent, very good, good = 0, fair, poor = 1
39.health now compared with 1 year ago	worse =1, better = 0
40.overnight hospital patient in last year	yes =1, no =0
41.times receive healthcare over past year	no=0, 1-4=0.5, ≥5 =1
42.number of prescription medicines taken	no =0, 1-4=0.5, ≥5 =1

**Physical Anthropometry**	
43.body mass index (kg/m^2)	<18.5, ≥30=1<br>≥25, <30=0.5<br>≥18.5,<25=0
**Laboratory values**	
44.glycohemoglobin (%)	0%-5.7%=0, >5.7%=1
45.red blood cell count (million cells/ul)	M: ≥4.7, <6.1=0, Other=1<br>F: ≥4.2,<5.4=0, Other =1
46.hemoglobin (g/dl)	M: ≥13.5, <18 =0, Other =1<br>F: ≥12, <16 =0, Other=1
47.red cell distribution width (%)	≥11.6, <14.6=0, Other=1
48.lymphocyte percent (%)	≥20, <40=0, Other=1
49.segmented neutrophils percent (%)	≥40, <80=0, Other=1

Supplementary Table S2. GWAS data source and specific information

Variable	Data sources	Phenotype Code	population	year	sample size	Number of SNPs
frailty	https://www.ebi.ac.uk/gwas/home	GCST90020053	Europe	2021	175,226	7,663,023
chest pain	https://www.nealelab.is/uk-biobank	2335	Europe	2018	501,260	13,770,979

Supplementary Table S3.Weighted baseline characteristics of participants

variables	Frailty Index			Frail group			Non-frail group		
	All (N=1019)	No Chest Pain (N=653) / Chest Pain (N=366)	p	All (N=642)	No Chest Pain (N=349) / Chest Pain (N=293)	P	All (N=377)	No Chest Pain (N=304) / Chest Pain (N=73)	P
**Age, years, mean (SD)**	64.18 (10.67)	64.95(10.34)/ 62.81(11.11)	0.014	64.05 (11.29)	65.12(10.97)/ 62.77(11.54)	0.002	64.41 (9.53)	64.76(9.59)/ 62.97(9.23)	0.338
**TC,mg/dL, mean (SD)**	183.43 (45.89)	183.71(47.34)/ 182.92(43.23)	0.525	180.93 (47.61)	179.96(49.82)/ 182.08(44.91)	0.41	187.68 (42.52)	188.01 (44.03)/ 186.29 (35.79)	0.75
**TG,mmol/ L, mean (SD)**	1.81 (2.45)	1.85(2.94)/ 1.73 (1.11)	0.148	1.93 (2.98)	2.06(3.90)/ 1.77(1.13)	0.065	1.60 (1.02)	1.61(1.03)/ 1.57(1.01)	0.749
**Frailty index mean (SD)**	0.26 (0.11)	0.23(0.10)/ 0.31(0.12)	<0.001	0.33 (0.09)	0.31(0.07)/ 0.35(0.10)	<0.001	0.15 (0.04)	0.14 (0.04)/ 0.15 (0.04)	0.672
**FG, mg/dL, mean (SD)**	140.47 (53.00)	139.22(50.81)/ 142.70 (56.70)	0.229	144.24 (56.50)	144.34(56.29)/ 144.12(56.85)	0.975	134.07 (45.80)	133.36 (43.02)/ 137.03 (56.15)	0.457
**Gender, N (%)**									
Female	531 (52.1)	332(50.8)/ 199(54.4)	0.131	376 (58.6)	204(58.5)/ 172(58.7)	0.561	155 (41.1)	128(42.1)/ 27(37.0)	0.833
Male	488 (47.9)	321(49.2)/ 167(45.6)		266 (41.4)	145(41.5)/ 121(41.3)		222 (58.9)	176(57.9)/ 46(63.0)	
**Race, N (%)**									
Mexican American	134 (13.2)	85(13.0)/ 49(13.4)	0.256	89 (13.9)	46(13.2)/ 43(14.7)		45 (11.9)	39(12.8)/ 6(8.2)	0.048
Non-Hispanic Black	247 (24.2)	165(25.3)/ 82(22.4)		174(27.1)	103(29.5)/ 71(24.2)		73 (19.4)	62(20.4)/ 11(15.1)	

Non-Hispanic White	433 (42.5)	266(40.7)/ 167(45.6)		278 (43.3)	145(41.5)/ 133(45.4)	0.898	155 (41.1)	121(39.8)/ 34(46.6)	
Other Hispanic	122 (12.0)	78(11.9)/ 44(12.0)		60 (9.3)	33(9.5)/ 27(9.2)		62 (16.4)	45(14.8)/ 17(23.3)	
Other Race - Including Multi-Racial	83 (8.1)	59(9.0)/ 24(6.6)		41 (6.4)	22(6.3)/ 19(6.5)		42 (11.1)	37(12.2)/ 5(6.8)	
**Education, N (%)**									
Less than 9th grade	159 (15.6)	108(16.5)/ 51(13.9)		110 (17.1)	65(18.6)/ 45(15.4)		49 (13.0)	43(14.1)/ 6(8.2)	
9-11th grade	188 (18.4)	125(19.1)/ 63(17.2)		96 (15.0)	50(14.3)/ 46(15.7)		92 (24.4)	75(24.7)/ 17(23.3)	
High school graduate	229 (22.5)	145(22.2)/ 84(23.0)	0.698	148 (23.1)	79(22.6)/ 69(23.5)	0.922	81 (21.5)	66(21.7)/ 15(20.5)	0.63
Some college or AA degree	159 (15.6)	101(15.5)/ 58(15.8)		110 (17.1)	59(16.9)/ 51(17.4)		49 (13.0)	42(13.8)/ 7(9.6)	
College graduate or above	284 (27.9)	174(26.6)/ 110(30.1)		178 (27.7)	96(27.5)/ 82(28.0)		106 (28.1)	78(25.7)/ 28(38.4)	
**Smoke, N (%)**									
No	499 (49.0)	336(51.5)/ 163(44.5)	0.35	309 (48.1)	180(51.6)/ 129(44.0)	0.346	190 (50.4)	156(51.3)/ 34(46.6)	0.773
Yes	520 (51.0)	317(48.5)/ 203(55.5)		333 (51.9)	169(48.4)/ 164(56.0)		187 (49.6)	148(48.7)/ 39(53.4)	
**Hypertension, N (%)**									
No	169 (16.6)	114(17.5)/ 55(15.0)	0.87	72 (11.2)	39(11.2)/ 33(11.3)	0.593	97 (25.7)	75(24.7)/ 22(30.1)	0.406

Yes	850 (83.4)	539(82.5)/ 311(85.0)		570 (88.8)	310(88.8)/ 260(88.7)		280 (74.3)	229(75.3)/ 51(69.9)	
**Alcohol, N (%)**									
No	346 (34.0)	237(36.3)/ 109(29.8)	0.564	231 (36.0)	139(39.8)/ 92(31.4)	0.233	115 (30.5)	98 (32.2)/ 17(23.3)	0.599
Yes	673 (66.0)	416(63.7)/ 257(70.2)		411 (64.0)	210(60.2)/ 201(68.6)		262 (69.5)	206(67.8)/ 56 (76.7)	
**Anti-hyperlipidemic, N (%)**									
No	266 (26.1)	184(28.2)/ 82(22.4)	0.547	134 (20.9)	75(21.5)/ 59(20.1)	0.803	132 (35.0)	109(35.9)/ 23(31.5)	0.819
Yes	753 (73.9)	469(71.8)/ 284(77.6)		508 (79.1)	274(78.5)/ 234(79.9)		245 (65.0)	195(64.1)/ 50(68.5)	
**Antidiabetic, N (%)**									
No	472 (46.3)	276(42.3)/ 196(53.6)	0.001	276 (43.0)	123(35.2)/ 153(52.2)	<0.001	196 (52.0)	153(50.3)/ 43(58.9)	0.518
Yes	547 (53.7)	377(57.7)/ 170(46.4)		366 (57.0)	226(64.8)/ 140(47.8)		181 (48.0)	151(49.7)/ 30(41.1)	
**Heart failure, N (%)**									
No	908 (89.1)	612(93.7)/ 296(80.9)	<0.001	536 (83.5)	309(88.5)/ 227(77.5)	0.001	372 (98.7)	303(99.7)/ 69(94.5)	0.445
Yes	111 (10.9)	41(6.3)/ 70(19.1)		106 (16.5)	40(11.5)/ 66(22.5)		5(1.3)	1(0.3)/ 4(5.5)	
**Coronary heart disease, N (%)**									

No	894 (87.7)	611(93.6)/ 283(77.3)	<0.001	535 (83.3)	316(90.5)/ 219(74.7)	0.001	359 (95.2)	295(97.0)/ 64(87.7)	0.088
Yes	125 (12.3)	42(6.4)/ 83(22.7)		107 (16.7)	33(9.5)/ 74(25.3)		18 (4.8)	9(3.0)/ 9(12.3)	
**Angina pectoris, N (%)**									
No	933 (91.6)	637(97.5)/ 296(80.9)	<0.001	561 (87.4)	333(95.4)/ 228(77.8)	<0.001	372 (98.7)	304(100.0)/ 68 (93.2)	0.001
Yes	86 (8.4)	16(2.5)/ 70(19.1)		81 (12.6)	16(4.6)/ 65(22.2)		5 (1.3)	0(0.0)/ 5 (6.8)	
**Heart attack, N (%)**									
No	902 (88.5)	621(95.1)/ 281(76.8)	<0.001	537 (83.6)	323(92.6)/ 214(73.0)	<0.001	365 (96.8)	298(98.0)/ 67(91.8)	0.193
Yes	117 (11.5)	32(4.9)/ 85(23.2)		105 (16.4)	26(7.4)/ 79(27.0)		12 (3.2)	6(2.0)/ 6(8.2)	
**Stroke, N (%)**									
No	922 (90.5)	605(92.6)/ 317(86.6)	0.033	560 (87.2)	309(88.5)/ 251(85.7)	0.515	362 (96.0)	296(97.4)/ 66(90.4)	0.144
Yes	97 (9.5)	48(7.4)/ 49(13.4)		82 (12.8)	40(11.5)/ 42(14.3)		15 (4.0)	8(2.6)/ 7(9.6)	
**Activities, N (%)**									
No	952 (93.4)	608(93.1)/ 344(94.0)	0.65	607 (94.5)	333(95.4)/ 274(93.5)	0.061	345 (91.5)	275(90.5)/ 70(95.9)	0.428
Yes	67 (6.6)	45(6.9)/ 22(6.0)		35 (5.5)	16(4.6)/ 19(6.5)		32 (8.5)	29(9.5)/ 3(4.1)	
**T2DM, N (%)**									

No	237 (23.3)	136(20.8)/ 101(27.6)	0.027	111 (17.3)	40(11.5)/ 71(24.2)	<0.001	126 (33.4)	96(31.6)/ 30(41.1)	0.463
Yes	782 (76.7)	517(79.2)/ 265(72.4)		531 (82.7)	309(88.5)/ 222(75.8)		251 (66.6)	208(68.4)/ 43(58.9)	
**BMI, N (%)**									
Normal Weight	136 (13.3)	96(14.7)/ 40(10.9)		59 (9.2)	35(10.0)/ 24(8.2)		77 (20.4)	61(20.1)/ 16(21.9)	
Obese	593 (58.2)	361(55.3)/ 232(63.4)	0.594	426 (66.4)	227(65.0)/ 199(67.9)	0.87	167 (44.3)	134(44.1)/ 33(45.2)	0.911
Overweight	290 (28.5)	196(30.0)/ 94(25.7)		157 (24.5)	87(24.9)/ 70(23.9)		133 (35.3)	109(35.9)/ 24(32.9)	
**PIR, N (%)**									
High Income	193 (18.9)	146(22.4)/ 47(12.8)		87 (13.6)	62(17.8)/ 25(8.5)		106 (28.1)	84(27.6)/ 22(30.1)	
Low Income	264 (25.9)	158(24.2)/ 106(29.0)	0.007	193 (30.1)	100(28.7)/ 93(31.7)	0.088	71 (18.8)	58(19.1)/ 13(17.8)	0.652
Middle Income	562 (55.2)	349(53.4)/ 213(58.2)		362 (56.4)	187(53.6)/ 175(59.7)		200 (53.1)	162(53.3)/ 38(52.1)	

PIR = poverty income ratio; BMI = body mass index; TC = total cholesterol; FG = fasting glucose; TG = triglyceride; T2DM = type 2 diabetes mellitus.

Supplementary Table S4. The variance inflation factor (VIF) for all independent variables included

Variable	Unstandardized Coefficients		Standardized Coefficients	t	Sig.	Collinearity Statistics	
	B	Standard Error	Beta			VIF	Tolerance
(Constant)	-0.143	0.175	-	-0.815	0.418	-	-
Gender	-0.057	0.022	-0.256	-2.638	0.010*	1.375	0.727
Age	0	0.001	-0.018	-0.184	0.854	1.325	0.755
Race	-0.019	0.011	-0.162	-1.744	0.085	1.269	0.788
Education	0.012	0.008	0.152	1.564	0.122	1.39	0.72
Alcohol	0.019	0.025	0.074	0.764	0.447	1.384	0.722
Antihyperlipidemic	0.028	0.034	0.079	0.823	0.413	1.365	0.733
Chest pain	0.035	0.024	0.149	1.469	0.146	1.506	0.664
Antidiabetic	0.022	0.03	0.099	0.728	0.469	2.686	0.372
Hypertension	0.055	0.029	0.183	1.901	0.061	1.35	0.741
Heart failure	0.109	0.044	0.233	2.465	0.016*	1.312	0.762
Angina pectoris	0.114	0.067	0.345	1.692	0.095	6.07	0.165
Heart attack	-0.005	0.056	-0.012	-0.081	0.936	3.01	0.332
Coronary heart disease	-0.01	0.058	-0.032	-0.168	0.867	5.125	0.195
Stroke	0.097	0.033	0.261	2.899	0.005**	1.189	0.841
Activities	0.005	0.043	0.013	0.127	0.899	1.428	0.7
Smoke	-0.036	0.021	-0.161	-1.685	0.096	1.338	0.747
T2DM	0.014	0.033	0.06	0.442	0.66	2.69	0.372
BMI	-0.018	0.017	-0.106	-1.053	0.296	1.482	0.675
PTR	0	0.014	0.002	0.023	0.981	1.668	0.6
TC	0	0	-0.066	-0.571	0.57	1.971	0.507

TG	-0.005	0.009	-0.066	-0.584	0.561	1.9	0.526
FG	0	0	-0.026	-0.255	0.8	1.565	0.639

PIR = poverty income ratio; BMI = body mass index; TC = total cholesterol; FG = fasting glucose; TG = triglyceride; T2DM = type 2 diabetes mellitus.

Supplementary Table S5. Sensitivity analysis comparing data before and after multiple interpolation

variables	After(N=1099)	Before(N=1019)	p
**Age, years, mean (SD)**	64.25 (10.63)	64.18 (10.67)	0.881
**frailty index,mean(SD)**	0.26 (0.12)	0.26 (0.11)	0.331
**FG,mg/dL,mean(SD)**	140.39 (53.15)	140.47 (53.00)	0.972
**TC,mg/dL,mean (SD)**	183.12 (45.92)	183.43 (45.89)	0.876
**TG,mmol/L,mean(SD)**	1.81 (2.38)	1.81 (2.45)	0.979
**Gender,N(%)**			0.945
Female	570 (51.9)	531 (52.1)	
Male	529 (48.1)	488 (47.9)	
**Race,N (%)**			0.970
Mexican American	140 (12.7)	134 (13.2)	
Non-Hispanic Black	281 (25.6)	247 (24.2)	
Non-Hispanic White	458 (41.7)	433 (42.5)	
Other Hispanic	130 (11.8)	122 (12.0)	
Other Race - Including Multi-Racial	90 (8.2)	83 (8.1)	
**Education,N (%)**			0.992
9-11th grade (Includes 12th grade with no diploma)	172 (15.7)	159 (15.6)	
College graduate or above	209 (19.0)	188 (18.4)	

High school graduate/GED or equivalent	243 (22.1)	229 (22.5)	
Less than 9th grade	165 (15.0)	159 (15.6)	
Some college or AA degree	310 (28.2)	284 (27.9)	
**Alcohol,N (%)**			0.902
No	377 (34.3)	346 (34.0)	
Yes	722 (65.7)	673 (66.0)	
**Anti-hyperlipidemic, N (%)**			0.997
No	288 (26.2)	266 (26.1)	
Yes	811 (73.8)	753 (73.9)	
**Chest pain, N (%)**			0.976
No	706 (64.2)	653 (64.1)	
Yes	393 (35.8)	366 (35.9)	
**Antidiabetic, N (%)**			0.705
No	499 (45.4)	472 (46.3)	
Yes	600 (54.6)	547 (53.7)	
**Hypertension,N (%)**			0.945
No	180 (16.4)	169 (16.6)	
Yes	919 (83.6)	850 (83.4)	
**Heart failure, N (%)**			1
No	979 (89.1)	908 (89.1)	
Yes	120 (10.9)	111 (10.9)	

**Coronary heart disease, N (%)**			0.960
No	966 (87.9)	894 (87.7)	
Yes	133 (12.1)	125 (12.3)	
**Angina pectoris, N (%)**			0.987
No	1005 (91.4)	933 (91.6)	
Yes	94 (8.6)	86 (8.4)	
**Heart attack, N (%)**			0.992
No	974 (88.6)	902 (88.5)	
Yes	125 (11.4)	117 (11.5)	
**Stroke, N (%)**			0.967
No	996 (90.6)	922 (90.5)	
Yes	103 (9.4)	97 (9.5)	
**Activities, N (%)**			1
No	1026 (93.4)	952 (93.4)	
Yes	73 (6.6)	67 (6.6)	
**Smoke, N (%)**			0.874
No	543 (49.4)	499 (49.0)	
Yes	556 (50.6)	520 (51.0)	
**T2DM, N (%)**			0.820
No	250 (22.7)	237 (23.3)	
Yes	849 (77.3)	782 (76.7)	
**BMI, N (%)**			0.953

Normal Weight	151 (13.7)	136 (13.3)	
Obese	640 (58.2)	593 (58.2)	
Overweight	308 (28.0)	290 (28.5)	
**PTR, N (%)**			0.913
High Income	215 (19.6)	193 (18.9)	
Low Income	287 (26.1)	264 (25.9)	
Middle Income	597 (54.3)	562 (55.2)	

PIR = poverty income ratio; BMI = body mass index; TC = total cholesterol; FG = fasting glucose; TG = triglyceride; T2DM = type 2 diabetes mellitus.

Supplementary Table S6. Unweighted logistic regression associations between frailty index、frail status and non-frail status and chest pain

Exposure	Model 1<br>OR (95%CI)<br>[P value]	Model 2<br>OR (95%CI)<br>[P value]	Model 3<br>OR (95%CI)<br>[P value]
**Frailty index**			
**Quartiles**			
Q1	Reference	Reference	Reference
Q2	2.14(1.40 - 3.25)<br>[$p < 0.001$]	2.26 (1.47 - 3.48)<br>[$p < 0.001$]	2.09 (1.31 - 3.35)<br>[$p = 0.002$]
Q3	3.18 (2.11 - 4.80)<br>[$p < 0.001$]	3.37 (2.20 - 5.16)<br>[$p < 0.001$]	2.64 (1.62 - 4.32)<br>[$p < 0.001$]
Q4	6.15 (4.09 - 9.25)<br>[$p < 0.001$]	6.80 (4.39 - 10.52)<br>[$p < 0.001$]	4.58 (2.70 - 7.77)<br>[$p < 0.001$]
**Frail status**			
**Quartiles**			
Q1	Reference	Reference	Reference
Q2	1.17(0.75 - 1.84)<br>[$p = 0.489$]	1.19(0.75 - 1.91)<br>[p = 0.461]	1.22(0.72 - 2.06)<br>[p = 0.455]
Q3	1.51(0.96 - 2.37)<br>[$p = 0.072$]	1.57(0.99 - 2.50)<br>[$p = 0.058$]	1.58(0.93 - 2.68)<br>[$p = 0.092$]
Q4	3.07(1.95 - 4.84)<br>[$p < 0.001$]	3.10(1.93 - 5.00)<br>[$p < 0.001$]	2.25(1.30 - 3.92)<br>[$p = 0.004$]
**non-frail status**			
**Quartiles**			
Q1	Reference	Reference	Reference
Q2	1.17 (0.56 - 2.46)<br>[p = 0.680]	1.13 (0.52 - 2.44)<br>[$p = 0.766$]	1.26 (0.53 - 2.98)<br>[$p = 0.606$]
Q3	1.01 (0.47 - 2.17)<br>[p = 0.974]	1.10 (0.49 - 2.43)<br>[$p = 0.823$]	1.21 (0.49 - 3.01)<br>[$p = 0.682$]
Q4	1.60 (0.78 - 3.27)<br>[p =0.197]	1.89 (0.88 - 4.05)<br>[$p = 0.102$]	2.05 (0.82 - 5.08)<br>[$p = 0.123$]

Model 1: Unadjusted model.

Model 2: Adjusted for age, sex, race, PIR, and education level.

Model 3: Adjusted for age, sex, race, PIR, education level, BMI, smoking status,

alcohol intake status, hypertension, TG, TC , FG , vigorous physical activity, heart failure, coronary heart disease, angina pectoris, heart attack, stroke, and T2DM, antihyperlipidemic and antidiabetic medications.

OR = odds ratio; CI = confidence interval; PIR = poverty income ratio; BMI = body mass index; TG = triglyceride; TC = total cholesterol; FG = fasting glucose; T2DM = type 2 diabetes mellitus.

Supplementary Table S7. Unweighted Logistic Regression Analyses of the Associations Between Frailty Index, Frailty Status, and Chest Pain (Based on Multiply Imputed Data)

Exposure	Model 1<br>OR (95%CI)<br>[P value]	Model 2<br>OR (95%CI)<br>[P value]	Model 3<br>OR (95%CI)<br>[P value]
**Frailty index**			
**Quartiles**			
Q1	Reference	Reference	Reference
Q2	1.72(0.89 - 3.32）<br>[p = 0.111]	1.73 (0.90 - 3.32)<br>[p = 0.107]	1.48(0.74 - 2.95)<br>[p = 0.277]
Q3	2.34 (1.20 - 4.59)<br>[p = 0.016]	2.27(1.17 - 4.41)<br>[p = 0.020]	1.90(0.96 - 3.78)<br>[p = 0.077]
Q4	5.22 (2.98 - 9.15)<br>[p < 0.001]	4.99(2.77 - 8.97)<br>[p < 0.001]	3.53(1.73 - 7.21)<br>[p = 0.002]
**Frail status**			
**Quartiles**			
Q1	Reference	Reference	Reference
Q2	1.21(0.70 - 2.09)<br>[p = 0.494]	1.15(0.68 - 1.94)<br>[p = 0.607]	1.35(0.78 - 2.36)<br>[p = 0.296]
Q3	1.31(0.80 - 2.16)<br>[p = 0.287]	1.27(0.78 - 2.05)<br>[p = 0.338]	1.36(0.82 - 2.26)<br>[p = 0.238]
Q4	3.01(1.63 - 5.55)<br>[p =0.001]	3.00(1.56 - 5.78)<br>[p =0.002]	2.20(1.10 - 4.43)<br>[p = 0.035]
**non-frail status**			
**Quartiles**			
Q1	Reference	Reference	Reference
Q2	2.24(0.86-5.85)<br>[p = 0.106]	2.06(0.80-5.30)<br>[p = 0.146]	3.63(0.88-14.95)<br>[p = 0.099]
Q3	1.54(0.48-4.89)<br>[p = 0.471]	1.43(0.49-4.19)<br>[p = 0.515]	1.78(0.53-5.97)<br>[p = 0.372]
Q4	1.85(0.64-5.36)<br>[p =0.266]	1.95(0.63-6.00)<br>[p = 0.255]	2.91(0.66-12.73)<br>[p = 0.182]

Model 1: Unadjusted model.

Model 2: Adjusted for age, sex, race, PIR, and education level.

Model 3: Adjusted for age, sex, race, PIR, education level, BMI, smoking status, alcohol intake status, hypertension, TG, TC , FG , vigorous physical activity, heart failure, coronary heart disease, angina pectoris, heart attack, stroke, and T2DM, antihyperlipidemic and antidiabetic medications.

OR = odds ratio; CI = confidence interval; PIR = poverty income ratio; BMI = body mass index; TG = triglyceride; TC = total cholesterol; FG = fasting glucose; T2DM = type 2 diabetes mellitus.

Supplementary Table S8. Characteristics of 31 SNPs used as instrumental variables for the effect of frailty on chest pain

SNP	Other_allele	Effect_allele	Beta	Se	Eaf	Pval	F
rs11245450	G	A	-0.0171	0.0034	0.4251	4.20E-07	25.29
rs117133873	C	A	0.037	0.007	0.0606	1.54E-07	27.94
rs11872713	C	T	-0.0244	0.0046	0.1554	8.92E-08	28.14
rs11951033	C	T	-0.0181	0.0035	0.3505	2.32E-07	26.74
rs12131852	G	C	0.0188	0.0037	0.711	2.66E-07	25.82
rs12441344	G	A	0.0214	0.0041	0.7906	1.36E-07	27.24
rs12739243	C	T	0.0242	0.004	0.7794	1.28E-09	36.60
rs1363103	C	T	0.0191	0.0034	0.62	2.23E-08	31.56
rs17144411	G	T	0.0217	0.0042	0.1869	3.19E-07	26.69
rs17379561	T	A	-0.0245	0.0047	0.8546	1.90E-07	27.17
rs2071207	C	T	0.0187	0.0033	0.522	1.47E-08	32.11
rs2396766	G	A	0.0201	0.0033	0.4725	1.22E-09	37.10
rs34045288	C	T	0.0179	0.0035	0.3357	3.29E-07	26.16
rs34651062	G	T	0.037	0.0073	0.058	3.42E-07	25.69
rs35096827	C	T	-0.03	0.0056	0.0987	8.58E-08	28.70
rs3959554	G	A	-0.0189	0.0034	0.5823	1.74E-08	30.90
rs4146140	C	T	-0.0198	0.0034	0.3811	6.83E-09	33.91
rs4952693	C	T	-0.0194	0.0034	0.3734	1.47E-08	32.56
rs56040623	C	T	-0.0239	0.0046	0.1498	2.47E-07	26.99
rs56299474	C	A	0.0241	0.0044	0.1733	3.94E-08	30.00
rs589292	C	T	-0.0181	0.0036	0.3133	3.89E-07	25.28
rs71460103	G	T	-0.0224	0.0042	0.1988	6.87E-08	28.44
rs755249	C	T	0.0203	0.0039	0.2318	2.21E-07	27.09
rs7603172	G	A	0.0193	0.0036	0.3022	7.25E-08	28.74
rs8089807	C	T	-0.0248	0.0043	0.1866	6.50E-09	33.26
rs8115058	G	A	0.0177	0.0034	0.626	2.32E-07	27.10
rs82334	C	A	0.0223	0.0035	0.6823	3.13E-10	40.60
rs9275160	G	A	0.0382	0.0035	0.3397	7.18E-28	119.12
rs9356282	C	T	0.0213	0.0039	0.2338	5.29E-08	29.83
rs9823373	C	T	-0.0203	0.0039	0.7587	1.37E-07	27.09
rs9894577	G	A	0.0179	0.0036	0.3148	5.00E-07	24.72

NOTE：$F = \beta^2/se^2$

Supplementary Table S9. Results of heterogeneity and multiplicity tests for MR

Exposes	Outcomes	Q-statistics				Pleiotropic test(mr_egger)		Pleiotropic test（mr_presso)	
		mr_egger	pval	IVW	pval	Egger _intercept	pval	Global Test RSSobs	Global Test Pvalue
Frailty	chest pain	23.75	0.74	25.81	0.68	0.00	0.16	27.46	0.87

IVW = inverse variance weighting;MR = Mendelian randomization.
