# Supplementary material for: Association between frailty and chest pain: Insights from the 2009 to 2018 NHANES cross-sectional analysis and Mendelian randomization
Source: Medicine (Baltimore). 2025 Sep 19;104(38):e44517. doi: 10.1097/MD.0000000000044517 (PMC12459587; doi:10.1097/MD.0000000000044517)
Supplement: Supplementary file 2 [file medi-104-e44517-s002.pdf]

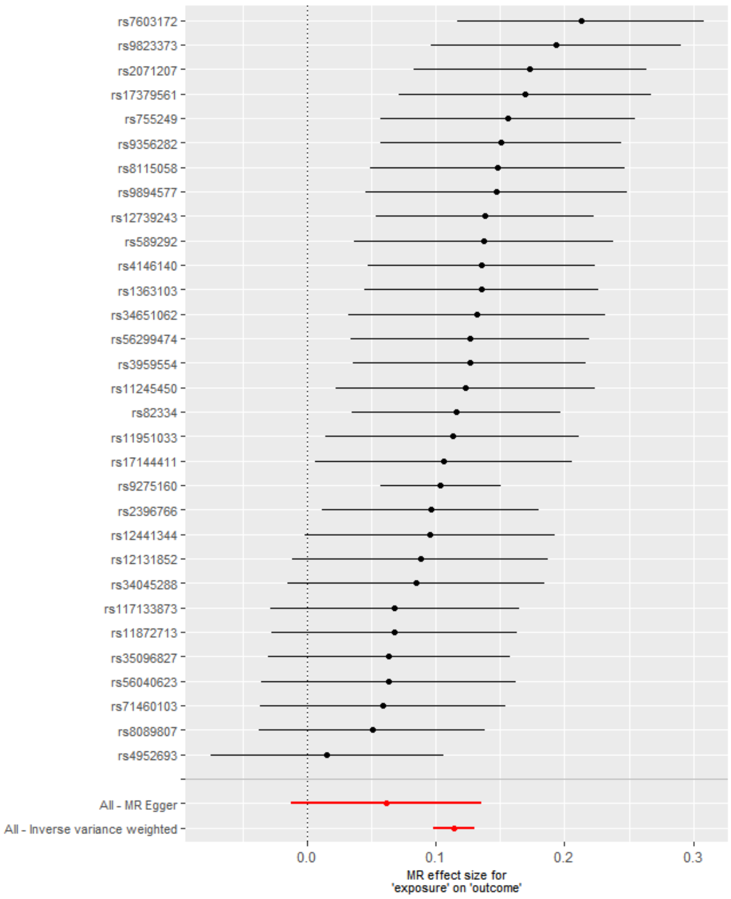
rs7603172
rs9823373
rs2071207
rs17379561
rs755249
rs9356282
rs8115058
rs9894577
rs12739243
rs589292
rs4146140
rs1363103
rs34651062
rs56299474
rs3959554
rs11245450
rs82334
rs11951033
rs17144411
rs9275160
rs2396766
rs12441344
rs12131852
rs34045288
rs117133873
rs11872713
rs35096827
rs56040623
rs71460103
rs8089807
rs4952693
All - MR Egger
All - Inverse variance weighted
0.0
0.1
0.2
0.3
MR effect size for
'exposure' on 'outcome'

(A)

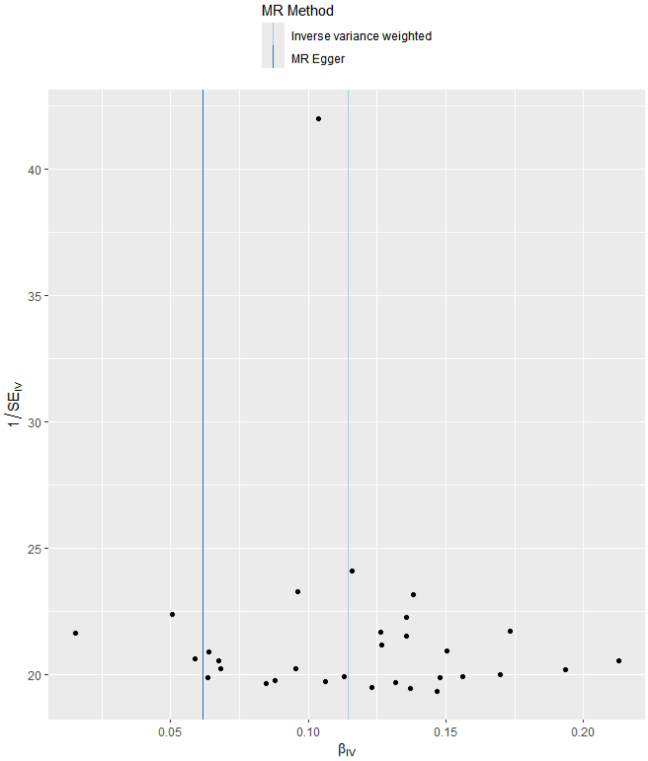
MR Method
Inverse variance weighted
MR Egger
40
35
30
25
20
1/SE_IV
0.05
0.10
0.15
0.20
β_IV

(B)

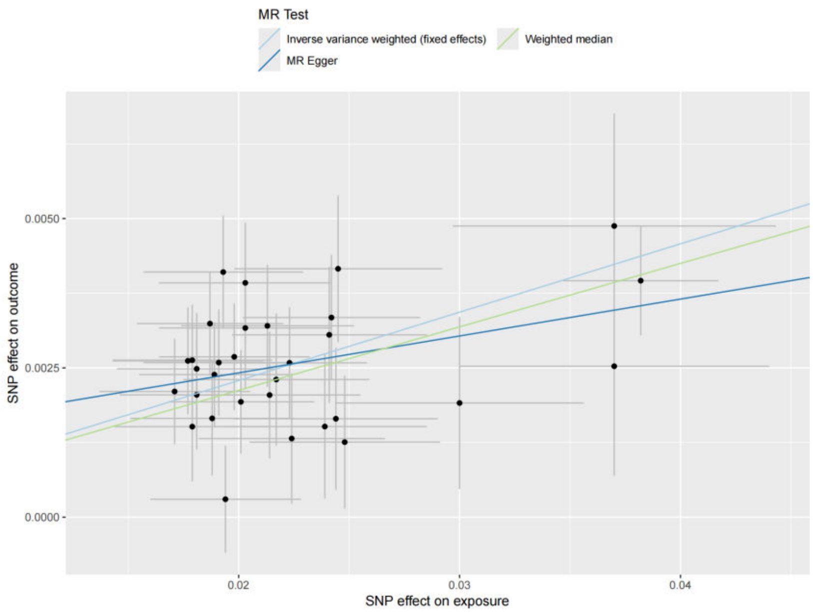

(C)

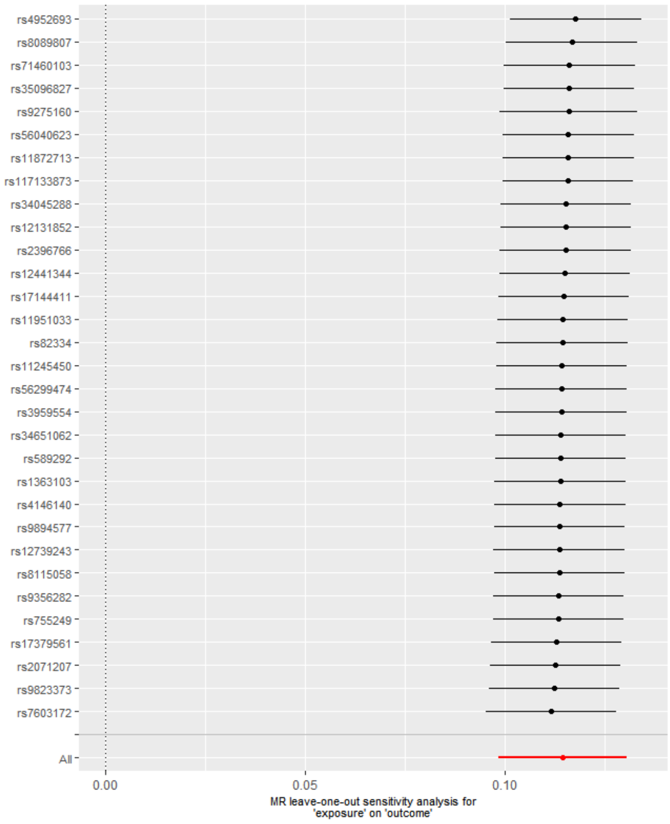

(D)

Supplementary Figure S1.Results of MR analysis:(A) Forest plot of the associations between frailty-associated SNPs and chest pain;(B) Funnel plot of the associations between frailty-associated SNPs and chest pain;(C) Scatterplot of the associations between frailty-associated SNPs and chest pain;(D) Leave-one-out analyses of the associations between frailty-associated SNPs and chest pain.
